# Physical Therapy Management for Delayed Diagnosis of Developmental Dysplasia of the Hip: A Case Report

**DOI:** 10.1155/crpe/5633998

**Published:** 2025-09-04

**Authors:** Kai-Yu Ho, Lisa Taylor, Katherine Joines

**Affiliations:** ^1^Department of Physical Therapy, University of Nevada, Las Vegas, Las Vegas, Nevada, USA; ^2^Clark County School District, Las Vegas, Nevada, USA

**Keywords:** case report, differential diagnosis, hip dysplasia, infant, physical therapy

## Abstract

**Background:** Delayed diagnosis of developmental dysplasia of the hip (DDH)—defined as detection after 8 weeks of age using physical examination, ultrasound, or X-ray—occurs in approximately 0.14%–0.26% of infants. This case report highlights the challenges of delayed DDH diagnosis and the role of physical therapy in rehabilitation.

**Case Report:** The patient, a firstborn Asian female, was born vaginally at 40 weeks gestation. Her early medical history included left muscular torticollis, asymmetric crying faces syndrome, and laryngomalacia. Parents observed asymmetric gluteal folds, but early physical examinations before 3 months showed negative Barlow and Ortolani tests, normal hip range of motion, and no motor impairments. Thus, ultrasound imaging was not deemed necessary in early infancy due to negative physical exam findings, the absence of classic presentations and signs, and a lack of major risk factors that would typically warrant further imaging evaluation. At 11 months, signs of reduced weight bearing and leg length discrepancy led to radiographic evaluation, revealing left DDH with subluxation. Treatment included closed reduction, 14 weeks in a Spica cast, 14 months of abduction bracing, and physical therapy. Following casting, the patient experienced hip stiffness, limited mobility, and muscle weakness. Physical therapy focused on restoring movement while ensuring joint stability. With weekly sessions, the patient showed significant progress, achieving independent walking at 19 months. Follow-up radiographs demonstrated gradual acetabular index improvement, approaching normal development by age 6 years and 9 months.

**Conclusion:** This case highlights the challenges of early DDH detection, the consequences of delayed diagnosis, and the vital role of physical therapy in postoperative recovery and functional development of children with DDH.

## 1. Introduction

Developmental dysplasia of the hip (DDH) is a condition characterized by improper alignment or abnormal growth of the femoral head and acetabulum, which, if left untreated, can lead to long-term functional limitations [1–4]. While early detection through newborn screening greatly enhances outcomes, delayed diagnoses—occurring beyond eight weeks after birth—remain a challenge due to subtle clinical presentations, variations and challenges in screening practices, and socioeconomic barriers [5–7]. The major risk factors for DDH include female sex, breech positioning, and a family history of DDH. The minor risk factors may include being a firstborn child, oligohydramnios, and the presence of comorbidities such as torticollis and clubfoot [8–10].

While physical examination techniques like the Barlow and Ortolani tests are essential for early infant screening, they have relatively low sensitivity and their accuracy decreases with age, which may result in missed diagnoses of some cases [2, 5, 11, 12]. Another challenge within the healthcare system is that at-risk infants may not receive necessary imaging studies, particularly when classic signs, symptoms, and major risk factors are absent or unclear, or when healthcare providers have limited time during patient visits [5–7, 13]. This lack of further imaging investigation can lead to missed or delayed diagnoses of DDH, which may later require invasive interventions such as open reduction and pelvic osteotomy—procedures that carry risks including avascular necrosis, sciatic nerve injury, and early-onset hip osteoarthritis [1].

Given these challenges, pediatric physical therapists play a vital role in detecting, differentiating, and managing DDH [9, 14], particularly in cases where early signs are missed and initial physical examinations yield negative results. With more frequent and prolonged contact during treatment sessions, physical therapists are well-positioned to identify subtle signs of DDH. In addition, as physical therapists regularly evaluate movement patterns and walking, they are likely to detect abnormalities in children with missed diagnoses who may start showing signs later, particularly as they begin weight bearing or walking. A recent scoping review highlighted the effectiveness of physical therapy in managing DDH, particularly in improving functional outcomes and reducing pain, underscoring the essential role of physical therapy in the rehabilitation of children with DDH [15].

This case report follows a child diagnosed with DDH at 11 months, illustrating how the diagnosis was initially missed and highlighting the difficulties of identifying and managing DDH beyond the newborn stage. Over the course of 6 years, the patient underwent several interventions, resulting in both radiographic and functional improvements. The case underscores the limitations of routine physical exam screenings and the consequences of delayed diagnosis. Through this report, we aim to raise clinical awareness of delayed DDH diagnoses and emphasize the critical role of physical therapy in both detection and rehabilitation.

## 2. Case Presentation

### 2.1. Birth and Medical History

The patient, a firstborn Asian female, was delivered vaginally at 40 weeks of gestation. Her 36-year-old mother underwent labor induction, culminating in a delivery assisted by vacuum extraction. The baby assumed a typical cephalic position with a birth weight of 3260 g. Apgar scores were 10 at both 1 and 5 mins after birth. Both the mother and the patient were discharged after a 3-day hospital stay (without intensive care unit admission). The patient did not need any respiratory support or phototherapy during their time in the nursery. Within the first month of life, the patient received diagnoses for multiple conditions, including left muscular torticollis, asymmetric crying faces syndrome, and laryngomalacia. In addition, at 9 months of age, bilateral esotropia was identified. There was no known family history of DDH.

### 2.2. Early Physical Therapy Intervention (1–10 Months)

To address torticollis, the patient began weekly physical therapy sessions at 1 month old as part of an early intervention program, continuing until 10 months. The comprehensive approach encompassed stretching for the left sternocleidomastoid muscle, strengthening for the right neck musculature, optimizing head and neck positioning, and modifying daily activities, home exercises, and educational guidance to the parents. Between 4 and 7 months, the patient also underwent cranial orthosis treatment for plagiocephaly.

Despite the mother noting asymmetric gluteal folds between the patient's legs, physical examinations by pediatricians and physical therapists during newborn screening and early infancy checkups before 3 months yielded negative results in Barlow and Ortolani tests. The assessments also showed normal and symmetric hip joint ranges of motion bilaterally, symmetric leg length (negative Galeazzi sign), and no motor milestone delays from 0 to 10 months. Consequently, pediatricians deemed imaging studies unnecessary, primarily due to the absence of physical examination abnormalities or urgent signs of DDH during this period, despite parents expressing concerns about uneven gluteal folds. The parents were assured that the hips were within the normal range, as asymmetrical gluteal folds may not be a primary indicator of DDH [16]. Nonetheless, the therapist maintained vigilance and continued monitoring for any potential signs of DDH, in alignment with current literature recommendations [17].

### 2.3. Detection of DDH (11 Months)

At 11 months, as the patient started pulling herself up to stand, the physical therapist noted reduced weight bearing on the left limb and a leg length discrepancy (shorter left limb). These signs raised concerns about the possibility of DDH. It is important to highlight that Barlow and Ortolani tests lose sensitivity in infants older than 3–6 months due to muscular growth [1]. Consequently, the therapist referred the case to an orthopedic surgeon for further evaluation, leading to an X-ray examination. For older infants, typically over 4–6 months, X-ray imaging is recommended for better interpretation of DDH due to femoral head ossification [1].

### 2.4. Radiographic Findings (11 Months)

**Figure 1:**
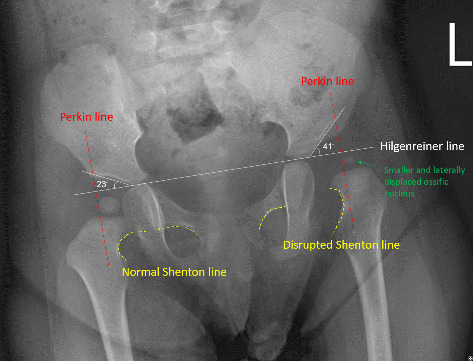
The X-ray taken at 11 months revealed disrupted Shenton line, increased acetabular index, subluxation of the left hip, and a small ossific nucleus of the capital femoral epiphysis in the left hip.

The X-ray taken at 11 months revealed abnormal findings in the patient's left hip (Figure 1). First, the Shenton line is typically a continuous curve along the inferior border of the superior pubic ramus and the inferomedial border of the femoral neck (see normal Shenton line in the right hip) [18]. In this case, disruption of the left Shenton line is observed, suggesting deviations from the normal hip joint alignment [18]. Second, the acetabular index is a crucial indicator for assessing acetabulum development, defined as the angle between the Hilgenreiner line and the line extending through to the lateral aspect of the acetabular roof [19]. The Hilgenreiner line, a horizontal reference, is drawn through the inferior aspect of both triradiate cartilages, the Y-shaped epiphyseal plate located at the junction of the ischium, ilium, and pubis in skeletally immature individuals [19]. The optimal acetabular index is recommended to be below 25° at 12 months, further reducing to less than 19–20° by 24 months [20–22]. A normal acetabular index indicates improved coverage of the acetabulum over the femoral head. In this case, an increased acetabular index was identified for the left hip (41°), contrasting with 23° in the right hip, suggesting a deviation from typical development of the left acetabulum. Third, the Perkin line and Hilgenreiner line act as references for identifying hip subluxation or dislocation. The Perkin line, perpendicular to the Hilgenreiner line, intersects the outermost point of the acetabular roof [22]. Typically, the ossific nucleus of the capital femoral epiphysis should be located in the inferomedial quadrant—inferior to the Hilgenreiner line and medial to the Perkin line [22] (see normal alignment in the right hip). However, in this case, the left ossific nucleus of the capital femoral epiphysis was displaced laterally to the Perkin line, indicating subluxation of the left hip joint. Fourth, the ossific nucleus of the capital femoral epiphysis is expected to display symmetry and uniform size on both sides [22]. In this patient, a reduction in size was observed in the left ossific nucleus, indicating an underdeveloped femoral head attributed to the malalignment of the left hip joint. Based on these radiographic presentations, a diagnosis of left DDH with subluxation was confirmed.

### 2.5. Closed Reduction (11 Months)

**Figure 2:**
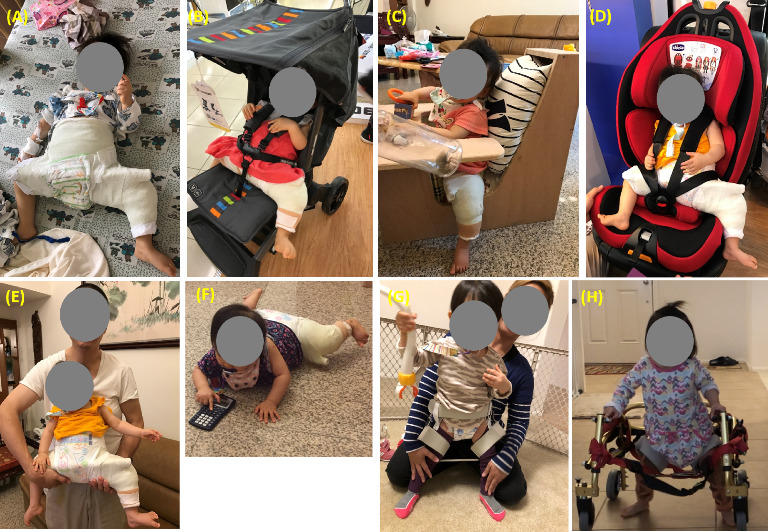
(A) Spica casting, (B) stroller, (C) table, (D) car seat utilized during Spica casting, (E) holding position during the casting phase, (F) tummy time with Spica cast, (G) abduction brace post-Spica casting removal, and (H) posterior walker employed in gait training.

A surgical management plan was implemented, which involved a closed reduction technique under general anesthesia. Once the hip joint was successfully reduced, the patient was placed in a Spica cast to ensure the proper alignment of the joint (Figure 2(A)). Fluoroscopy was performed during and after the surgical procedure to further verify the success of the joint reduction and Spica casting application.

Casting: 11 Months–1 Year and 2 Months and Bracing: 1 Year and 2 Months–2 Year and 4 Months.

The Spica casting period lasted for 14 weeks, with one cast change occurring at the 7-week mark. This duration is consistent with literature recommendations, which typically suggest 3-4 months of Spica casting following the reduction procedure [22]. This cast change allowed for re-evaluation of the joint alignment with the necessary hip position adjustments to be made during Spica casting. Hip positioning during Spica casting is individualized, with a range typically spanning from 90° to 100° of flexion and 45° to 60° of abduction. This positioning is aimed at achieving stable reduction while minimizing the risk of avascular necrosis [23]. As illustrated in Figure 2(A), during the initial 7 weeks, the surgeon positioned the patient with 90° of hip flexion and 90° of hip abduction. In the subsequent phase (8–14 weeks), as the joint stability improved during the first phase, the child was placed with 70° of hip flexion and 60° of hip abduction. In this case, due to the severity of hip instability as assessed by the surgeon, the hips were positioned at a relatively higher abduction angle throughout the Spica casting period.

**Figure 3:**
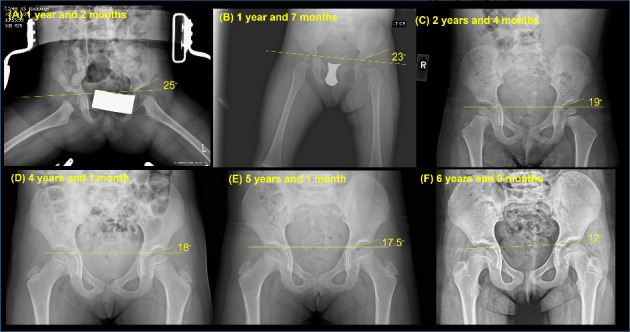
X-rays of the hip joints taken at (A) 1 year and 2 months, (B) 1 year and 7 months, (C) 2 years and 4 months, (D) 4 years and 1 month, (E) 5 years and 1 month, and (F) 6 years and 9 months.

Following the completion of the 14 weeks of Spica casting treatment, an improvement was observed in the hip joint alignment, along with a reduced acetabular index measuring 25° (Figure 3(A)). To maintain the achieved alignment, the patient began abduction bracing therapy, which involved wearing a brace that kept the hips abducted at approximately 45° (Figure 2(G)). The custom-made hip abduction brace featured hinges on both sides, allowing for 90° of flexion and 20° of extension in the hip joints during daily activities. This brace was designed to inhibit hip adduction while facilitating functional movements in daily life. It was consistently worn throughout the day from 1 year and 2 months to 2 years and 4 months, maximizing the advantages of maintaining proper joint positioning.

### 2.6. Physical Therapy Intervention During Spica Casting (11 Months–1 Year and 2 Months)

The therapist offered monthly sessions to support the family during Spica casting phase, addressing challenges, providing education on assistive tool techniques, guiding exercises, and refining holding and positioning methods. Particular emphasis was placed on assisting families in the careful selection of suitable tables, chairs, strollers, and car seats capable of meeting the unique positioning requirements imposed by the cast (Figures 2(B), 2(C), and 2(D)). Parents were also guided to relevant recommendations provided by the International Hip Dysplasia Institute to ensure safe and effective positioning [24]. In addition, the therapist provided resources and contacts for equipment rental or loan programs, offering families cost-effective solutions for obtaining the required assistive tools during the casting stage.

To address the constraints imposed by the cast, regular position changes and proper holding techniques were implemented to minimize the risk of immobility-related complications [25]. This included the strategic placement of pillows to facilitate the transition of the trunk between a fully supine position and a semiside lying position during sleep. When holding the patient, the focus was on providing support from the bottom of the cast rather than gripping the armpits, aiming to avoid potential spinal injuries from the cast's weight (Figure 2(E)). In addition, the family received guidance on incorporating tummy time into the patient's routine to enhance arm and trunk strength maintenance through crawling exercises (Figure 2(F)).

### 2.7. Physical Therapy Management After Removal of Spica Casting (1 Year and 2 Months–2 Years and 1 Month)

After Spica casting removal at 1 year and 2 months, stiffness and restricted hip joint range of motion were observed, with limited hip flexion (0–70°) and soreness upon movement. Weakness and inadequate control of lower extremity muscles were also noted, leading to trunk and hip wobbling during supported standing. The patient demonstrated significant gross motor delays, evident through a score of 0 in the “gross motor” category on the 14-month Ages & Stages Questionnaires (ASQ-3). Specifically, she struggled with skills such as maintaining a standing position while holding hands, taking consecutive steps forward while maintaining a grip, transitioning from sitting on the floor to standing, climbing onto furniture/large objects, performing squats or bending over and then returning to a standing position without assistance, and achieving independent walking.

Throughout the postcasting physical therapy sessions, a customized physical therapy plan and home exercise program (HEP) were consistently implemented. The family also received education on the proper utilization of the abduction brace and assistive devices. In addition, the physical therapist closely monitored for signs of avascular necrosis, including thigh pain and deteriorating hip range of motion, which can arise from compromised vascular circulation of the femoral head due to the elevated hip abduction and flexion angles utilized during the Spica casting period [23, 26]. This individualized approach is supported by previous studies that recognize the wide variability in clinical presentations and functional needs among children with DDH [15, 27–31]. Overall, the primary goals of physical therapy in managing DDH are to improve joint range of motion and muscular strength and to promote age-appropriate functional movement [15, 27–31]. Physical therapy sessions also included ongoing assessment of the patient's adherence to the HEP and monitoring for any challenges, adverse events, or unanticipated responses to ensure the intervention was well tolerated.

Following Spica casting removal, weekly physical therapy interventions were implemented with the goals of restoring hip joint range of motion within permissible limits while in the brace, improving muscle strength and control and attaining gross motor milestones such as crawling, sitting, standing, and walking. Initial exercises focused on enhancing lower extremity movement and muscle control, incorporating activities such as supported standing and marching while wearing the abduction brace. The goal was to promote natural movements while ensuring the maintenance of joint stability achieved during the Spica casting period (Figure 2(G)). As the patient progressed, activities like crawling with alternating legs, transitioning from sitting to standing, climbing, and squats were introduced. Adjustments (e.g., less support) were made based on progress, and guidance on using a posterior walker for independent standing and walking enhancement was provided at 1 year and 4 months (Figure 2(H)).

A significant milestone was achieved at 1 year and 7 months, as the patient independently walked without assistive devices, accompanied by an improved acetabular index of 23° (Figure 3(B)). Between 1 year and 8 months and 2 years and 1 month, the frequency of physical therapy sessions was reduced to twice a month, with a focus on mastering weight-bearing activities such as running, walking upstairs/downstairs with minimal support, kicking a ball, and jumping/hopping. The 20-month ASQ-3 assessment at 1 year and 8 months and the 24-month ASQ-3 assessment at 2 years revealed a gross motor score of 55 out of 60, indicating typical motor development for her age [32]. With significant milestones achieved, the patient was discharged from physical therapy at 2 years and 1 month.

### 2.8. Radiographic Follow-Ups (2 Years and 4 Months–6 Years and 9 Months)

The anterior–posterior view of the hip joints at 2 years and 4 months revealed a reduced acetabular index of 19°, indicating nearly age-appropriate acetabular development (Figure 3(C)). Consequently, the bracing therapy was discontinued. Following this, there were no specific restrictions on sports and activity participation. However, it is essential to note that hip adduction, such as “W” sitting, is still discouraged to maintain optimal hip alignment. The patient's acetabular index showed further improvement to 18° at 4 years and 1 month, subsequently to 17.5° at 5 years and 1 month, and 12° at 6 years and 9 months (Figures 3(D), 3(E), and 3(F)). Given that the left hip's development aligned with that of the right hip at 6 years and 9 months, the orthopedic surgeon advised scheduling the subsequent follow-up 5 years later, when the patient is approximately 11-12 years old. This recommendation was based on the favorable outcomes achieved at 6 years and 9 months and the minimal likelihood of a DDH recurrence.

## 3. Discussion

It is important to acknowledge that healthcare providers encounter a substantial challenge as cases of DDH may not exhibit all the classic features and/or lack clear physical signs, potentially leading to delayed diagnoses. For instance, this patient presented several risk factors and signs associated with DDH in infancy, including torticollis, being the firstborn child, Asian female, and asymmetrical gluteal folds. However, the patient exhibited negative results in key physical examinations, including Barlow and Ortolani tests, along with a normal Galeazzi sign and symmetric hip joint ranges of motion bilaterally within the first 3 months, complicating the diagnostic process.

As DDH can remain undiagnosed during early infancy, it often becomes more apparent when patients engage in weight-bearing activities such as standing and walking, exhibiting signs of limping, Trendelenburg sign, or other gait abnormalities, particularly in cases of unilateral DDH [1, 33]. In bilateral DDH, children may display symmetrical but reduced abduction during walking or adopt a waddling gait pattern [1]. In this particular case, the physical therapist identified reduced weight bearing and shorter limb length on the left at 11 months, prompting subsequent radiographic examinations and ultimately resulting in the diagnosis of DDH. These experiences highlight the need for physical therapists to stay vigilant and consistently monitor cases at risk of DDH.

In this case, despite the delayed diagnosis, the patient achieved positive outcomes through a less invasive approach, involving closed reduction followed by casting/bracing and subsequent physical therapy. Physical therapy plays a pivotal role in managing conditions postsurgery treatments for DDH, both during and after Spica casting. Throughout the Spica casting period, the therapist is tasked with evaluating the patient's home environment, making necessary modifications to accommodate specific needs, and designing a HEP. Post-Spica casting, children often present with limited hip joint motion, weakness, and poor lower extremity muscle control due to prolonged casting and immobilization [30]. The main goal is to restore age-appropriate functions, considering the child's age, prior activity levels, and hip joint intervention types [15, 27–31]. Overall, it is important to maintain proper hip positions and stability during therapy and at home, emphasizing caregiver education. Furthermore, providing education to families on the proper use of assistive devices following Spica casting and the utilization of abduction braces, commonly prescribed after surgical intervention for DDH, is crucial for physical therapists [34].

## 4. Conclusion

In this comprehensive case report, we detailed information about the disease progression, accompanied by a series of imaging findings for a case with a delayed diagnosis of DDH. Due to the late diagnosis, the patient underwent a closed reduction procedure, followed by Spica casting and prolonged bracing therapy to restore and sustain optimal joint alignment. Physical therapy played a crucial role in managing this DDH case. Timely detection of DDH signs and effective management of motor delays following the casting phase were critical in ensuring optimal outcomes for the patient.

## 5. Parent's Perspective

As a parent, hearing that our child had a delayed DDH was both confusing and frightening. What stood out to me was how the physical therapist became a crucial part of our journey—not just in recovery but in recognizing that something was not quite right in the first place. At 11 months, the therapist noticed subtle differences in our child's movement that we and even earlier screenings had missed. That insight led to a diagnosis we might not have, otherwise, received until much later. From there, the physical therapy team guided us through every step—teaching us exercises, helping us adjust to life with a Spica cast, and supporting our child's development as she began walking. Their knowledge, patience, and attention made all the difference. I truly believe that without physical therapy, we would have been facing a much harder road.

## Data Availability

The data that support the findings of this study are available from the corresponding author upon reasonable request.

## References

[1] Sioutis S., Kolovos S., Papakonstantinou M. E., Reppas L., Koulalis D., Mavrogenis A. F. (2022). Developmental Dysplasia of the Hip: A Review. *Journal of Long-Term Effects of Medical Implants*.

[2] US Preventive Services Task Force (2006). Screening for Developmental Dysplasia of the Hip: Recommendation Statement. *Pediatrics*.

[3] Pun S. (2016). Hip Dysplasia in the Young Adult Caused by Residual Childhood and Adolescent-Onset Dysplasia. *Current Reviews in Musculoskeletal Medicine*.

[4] Schmitz M. R., Murtha A. S., Clohisy J. C., Group A. S. (2020). Developmental Dysplasia of the Hip in Adolescents and Young Adults. *Journal of the American Academy of Orthopaedic Surgeons*.

[5] Mahan S. T., Katz J. N., Kim Y. J. (2009). To Screen or Not to Screen? A Decision Analysis of the Utility of Screening for Developmental Dysplasia of the Hip. *Journal of Bone and Joint Surgery American Volume*.

[6] Lindberg A. W., Bompadre V., Satchell E. K., Larson A. C. R., White K. K. (2017). Patient Factors Associated With Delayed Diagnosis of Developmental Dysplasia of the Hip. *Journal of Children’s Orthopaedics*.

[7] Murgai R. R., Harris L. R., Choi P. D., Goldstein R. Y. (2019). Socioeconomic Risk Factors for Poor Outcomes of Developmental Dysplasia of the Hip. *The Journal of Pediatrics*.

[8] Gkiatas I., Boptsi A., Tserga D., Gelalis I., Kosmas D., Pakos E. (2019). Developmental Dysplasia of the Hip: A Systematic Literature Review of the Genes Related With Its Occurrence. *EFORT Open Reviews*.

[9] de Hundt M., Vlemmix F., Bais J. M. (2012). Risk Factors for Developmental Dysplasia of the Hip: A Meta-Analysis. *European Journal of Obstetrics & Gynecology and Reproductive Biology*.

[10] Committee on Quality Improvement Subcommittee on Developmental Dysplasia of the Hip (2000). Clinical Practice Guideline: Early Detection of Developmental Dysplasia of the Hip. *Pediatrics*.

[11] Green N. E., Griffin P. P. (1982). Hip Dysplasia Associated With Abduction Contracture of the Contralateral Hip. *The Journal of Bone and Joint Surgery*.

[12] Chavoshi M., Soltani G., Shafiei Zargar S., Wyles C. C., Kremers H. M., Rouzrokh P. (2022). Diagnostic Performance of Clinical Examination Versus Ultrasonography in the Detection of Developmental Dysplasia of Hip: A Systematic Review and Meta-Analysis. *The Archives of Bone and Joint Surgery*.

[13] Shaw B. A., Segal L. S., Otsuka N. Y. (2016). Evaluation and Referral for Developmental Dysplasia of the Hip in Infants. *Pediatrics*.

[14] Vasilcova V., AlHarthi M., AlAmri N. (2022). Developmental Dysplasia of the Hip: Prevalence and Correlation With Other Diagnoses in Physiotherapy Practice-A 5-Year Retrospective Review. *Children*.

[15] Sharma A., Vats S., Gupta R. (2022). Effectiveness of Physiotherapy Intervention in Managing Patient’s Developmental Dysplasia of the Hip: A Scoping Review. *SN Comprehensive Clinical Medicine*.

[16] Kang M. S., Han G. W., Kam M., Park S. S. (2019). Clinical Significance of Asymmetric Skin Folds in the Medial Thigh for the Infantile Screening of Developmental Dysplasia of the Hip. *Pediatrics & Neonatology*.

[17] Palisano R., Orlin M., Vander Linden D. (2024). *Campbell’s Physical Therapy for Children*.

[18] Rhee P. C., Woodcock J. A., Clohisy J. C. (2011). The Shenton Line in the Diagnosis of Acetabular Dysplasia in the Skeletally Mature Patient. *Journal of Bone and Joint Surgery*.

[19] Sherman B., Lalonde F. D., Schlechter J. A. (2021). Measuring the Acetabular Index: An Accurate and Reliable Alternative Method of Measurement. *American Journal of Roentgenology*.

[20] Tönnis D. (1976). Normal Values of the Hip Joint for the Evaluation of X-Rays in Children and Adults. *Clinical Orthopaedics and Related Research*.

[21] Venkatadass K., Durga Prasad V., Al Ahmadi N. M. M., Rajasekaran S. (2022). Pelvic Osteotomies in Hip Dysplasia: Why, When and How?. *EFORT Open Reviews*.

[22] Nandhagopal T., De Cicco F. L. (2022). *Developmental Dysplasia of the Hip*.

[23] Li Y., Zhou Q., Liu Y. (2019). Closed Reduction and Dynamic Cast Immobilization in Patients with Developmental Dysplasia of the Hip Between 6 and 24 Months of Age. *European Journal of Orthopaedic Surgery and Traumatology*.

[24] Institute I. H. D. (2025). Understanding Hip Dysplasia—Infant & Child—Other Resources for Parents. https://hipdysplasia.org/infant-child/other-resources-for-parents/.

[25] Nie A. M., Delmore B. (2025). Hospitalized Pediatric Patients: Risk Factors Related to the Development of Immobility-Related and Medical Device-Related Pressure Injuries. *Advances in Skin & Wound Care*.

[26] Çitlak A., Şener M. (2013). The Incidence of Avascular Necrosis of the Femoral Head Changes With the Hip Abduction Angle in the Hip Spica in Treatment of Developmental Dislocation of the Hip. *Journal of Pediatric Orthopaedics B*.

[27] Marinela R. (2013). Early Physical Therapy Intervention in Infant Hip Dysplasia. *Procedia—Social and Behavioral Sciences*.

[28] Giorgi V., Apostolo G., Bertele L. (2024). Treatment of Developmental Hip Dysplasia With Manual Therapy Following Pavlik Harness Failure: A Case Report With Long-Term Follow-Up. *Journal of Manual & Manipulative Therapy*.

[29] Vieira Tavares B., Brandão Amorim P., Vicente Lopes L., Júlia Pereira Sena M., Victor Soares de Aguilar J. (2023). Influence of Physiotherapy Treatment for Developmental Dysplasia of the Hip After Use of the Pavlik Suspension. *International Seven Journal of Health Research*.

[30] Alanazi H., Almalik F., Alanazi N., Alhussainan T. (2020). Relapsed Hip Stiffness After Recovery of Range of Motion in a Hip Treated for Developmental Dysplasia of the Hip? Think Again: A Case Report. *International Journal of Surgery Case Reports*.

[31] Baheti N. C., Phansopkar P. (2024). Impact of Early Rehabilitation in a Four-Year-Old Patient With Developmental Dysplasia of the Hip: A Case Report. *Cureus*.

[32] Agarwal P. K., Xie H., Sathyapalan Rema A. S. (2020). Evaluation of the Ages and Stages Questionnaire (ASQ 3) as a Developmental Screener at 9, 18, and 24 Months. *Early Human Development*.

[33] Pandey R. A., Johari A. N. (2021). Screening of Newborns and Infants for Developmental Dysplasia of the Hip: A Systematic Review. *Indian Journal of Orthopaedics*.

[34] Merchant R., Singh A., Dala-Ali B., Sanghrajka A. P., Eastwood D. M. (2021). Principles of Bracing in the Early Management of Developmental Dysplasia of the Hip. *Indian Journal of Orthopaedics*.

